# Modulating Motor Learning through Transcranial Direct-Current Stimulation: An Integrative View

**DOI:** 10.3389/fpsyg.2016.01981

**Published:** 2016-12-23

**Authors:** Claudia Ammann, Danny Spampinato, Javier Márquez-Ruiz

**Affiliations:** ^1^Department of Physical Medicine and Rehabilitation, Johns Hopkins Medical InstitutionBaltimore, MD, USA; ^2^Division of Neurosciences, Pablo de Olavide UniversitySeville, Spain

**Keywords:** transcranial electrical stimulation, tDCS, motor learning, non-invasive brain stimulation, plasticity, skill learning, motor adaptation, use-dependent learning

## Abstract

Motor learning consists of the ability to improve motor actions through practice playing a major role in the acquisition of skills required for high-performance sports or motor function recovery after brain lesions. During the last decades, it has been reported that transcranial direct-current stimulation (tDCS), consisting in applying weak direct current through the scalp, is able of inducing polarity-specific changes in the excitability of cortical neurons. This low-cost, painless and well-tolerated portable technique has found a wide-spread use in the motor learning domain where it has been successfully applied to enhance motor learning in healthy individuals and for motor recovery after brain lesion as well as in pathological states associated to motor deficits. The main objective of this mini-review is to offer an integrative view about the potential use of tDCS for human motor learning modulation. Furthermore, we introduce the basic mechanisms underlying immediate and long-term effects associated to tDCS along with important considerations about its limitations and progression in recent years.

## Introduction

Motor learning entails improving motor actions through practice (Willingham, 1998; Dayan and Cohen, 2011; Wolpert et al., 2011). We make use of this ability when acquiring new motor skills and when adapting our movements to account for predictable changes to our environment. Motor learning plays a critical role in acquiring the motor actions necessary for high-performance sports (Nielsen and Cohen, 2008) and for motor recovery after brain lesions (Kitago and Krakauer, 2013). Applying weak direct current through the scalp induces polarity-specific changes in the excitability of cortical neurons (Nitsche et al., 2008; Brunoni et al., 2012). This effect of transcranial direct-current stimulation (tDCS) was first demonstrated in the human motor cortex (Nitsche and Paulus, 2000, 2001), but has also been described for other brain regions such as visual (Antal et al., 2001, 2004), somatosensory (Rogalewski et al., 2004; Dieckhöfer et al., 2006), prefrontal (Fregni et al., 2005; Mulquiney et al., 2011) and cerebellar cortices (Galea et al., 2009; Grimaldi et al., 2014). The modulatory effects and simplicity of tDCS have caught the attention of both basic and clinical neuroresearchers for its potential to modulate motor learning (Lang et al., 2003; Nitsche et al., 2003; Antal et al., 2004; Reis et al., 2008; López-Alonso et al., 2015). Most studies using tDCS deliver a low-current intensity (1–2 mA) between two rubber electrodes (25–35 cm^2^) placed on the scalp for 10–20 min (Stagg and Nitsche, 2011). For this montage, the stimulating electrode is placed over the region of interest while the reference electrode is placed over either the contralateral supraorbital, the mastoid or shoulder. Following this procedure, researchers have utilized tDCS to enhance motor learning in healthy individuals (Reis et al., 2008) and for motor recovery due to brain lesions or pathological states linked to motor deficits (Demirtas-Tatlidede et al., 2012; Grimaldi et al., 2014). tDCS has also been proposed to improve motor capacities and muscle endurance of high-performance sport athletes (Cogiamanian et al., 2007; Banissy and Muggleton, 2013; Williams et al., 2013). Although tDCS application in the motor domain is vast, the main objective of this review is to offer an integrative view of the main findings from studies using cerebral and cerebellar tDCS application in healthy human participants.

## Basic mechanisms underlying tDCS

Although there is increasing interest for using tDCS as a non-invasive neuromodulation technique, little is known about the molecular and/or cellular mechanisms underlying its effects (Márquez-Ruiz et al., 2012). Since Nitsche and Paulus (2000) described the impact of transcranial low current over the human primary motor cortex (M1), excitatory/inhibitory effects have been broadly associated to anodal/cathodal current stimulation, respectively. However, the net effect of tDCS depends on the stimulated brain region (Dieckhöfer et al., 2006), the number of tDCS sessions (Monte-Silva et al., 2013), the applied current intensity (Batsikadze et al., 2013), and the brain state (Silvanto and Pascual-Leone, 2008; Krause and Cohen Kadosh, 2014) among other parameters. To understand the physiological mechanisms underlying these effects, it is important to disassociate: a) the immediate tDCS effects observed in cells exposed to simultaneous exogenous electrical fields and b) effects mediated by protein modifications requiring longer stimulation periods, lasting for several minutes after tDCS application. The immediate effects are elicited when an external electric field causes displacement of intracellular ions, thus altering the internal charge distribution and modifying the neuronal membrane potential (Ruffini et al., 2013; Márquez-Ruiz et al., 2014). Moreover, animal studies have shown both neuronal morphology (Radman et al., 2009) and axonal orientation (Kabakov et al., 2012) are critical to consider when explaining tDCS-induced responses, since the maximal effects occur when electric fields are applied parallel to the somato-dendritic axis (Bikson et al., 2004). Beyond these somatic changes, animal studies have also demonstrated the importance of presynaptic effects during current application (Kabakov et al., 2012; Márquez-Ruiz et al., 2012; Bikson et al., 2013). The long-term effects, measured indirectly in human studies (recording motor evoked potentials, MEPs, elicited by transcranial magnetic pulses over M1) are mediated by N-methyl-D-aspartate (NMDA) and γ-aminobutyric acid type A (GABA_A_) receptors (see for review Stagg and Nitsche, 2011). Animal studies have confirmed the involvement of NMDA receptors and brain-derived neurotrophic factor (BDNF) (Fritsch et al., 2010) for the long-term effects observed after anodal direct-current stimulation (atDCS), and adenosine A1 receptors (Márquez-Ruiz et al., 2012) after cathodal direct-current stimulation (ctDCS).

## Modulating motor learning processes through tDCS

Motor learning encompasses various forms of learning, including, but not exclusive to error-based, reinforcement, use-dependent plasticity, and cognitive strategies (Krakauer and Mazzoni, 2011), each likely involving different neuronal substrates. It becomes more complicated given that these forms of learning likely all contribute to the learning process when acquiring a new skill (Kitago and Krakauer, 2013). Therefore, for better comprehensibility, we grouped publications based on different motor learning paradigms and not the different forms of learning, to explore the impact of tDCS on specific motor behaviors (see Table 1). We included adaptation, skill, and use-dependent repetition (i.e., repeated practice of simple movements) tasks. Undoubtedly, the number of positive findings described below, highlight the potential of tDCS for (1) modulating new behavior acquisition and retention, (2) identifying the underlying learning processes, and (3) studying the role of different brain regions.

**Table 1:** **A list of studies performed in healthy subjects integrating motor learning paradigms with transcranial direct current stimulation (tDCS) interventions**.

**Authors**	**Motor paradigm**	**Outcome measure**	**ROI**	**Moment of stimulation**	**Stim. site referring to performing site**	**Electrode montage**	**Parameters**	***J* mA/cm^2^**	**Groups**	**Key findings**
**SKILL LEARNING PARADIGMS**										
Nitsche et al., 2003	SRTT *right hand*	RTs of each block were divided by the RTs of block one	M1 PM lPFC mPFC	During learning	CL	M1 *Active:* C3 *Ref:* SO area PM *Active*: 2 cm forward, 2 cm to midline from M1 *Ref*: SO area Lateral PFC: *Active:* 5 cm anterior to C3 *Ref:* C4 Medial PFC: *Active:* SO area *Ref:* C4	1 mA, 35 cm^2^, 15 min	0.029	Anodal, cathodal, sham (crossover)	Improved acquisition and early retention with atDCS; no effects on remaining cortices
Kang and Paik, 2011	SRTT *right hand*	*Motor performance* = ratios of RTs in sequenced and random blocks	M1	During learning	CL	Uni-tDCS *Active:* C3 *Ref:* right SO area Bi-tDCS *Active*: C3 *Ref:* C4	2 mA, 25 cm^2^, 20 min	0.08	Anodal Uni-tDCS, Bi-tDCS, Sham (crossover)	No significant difference between Uni-tDCS and Bi-tDCS, in terms of performance. tDCS led to greater retention (24 h) than sham
Kantak et al., 2012	SRTT *left hand*	*Motor performance* = difference in mean RT between sequenced and random trials	M1 PMd	During learning	CL	M1 *Active:* FDI hotspot (TMS) *Ref:* left SO area PMd *Active:* 3 cm anterior, 1 cm medial to hotspot *Ref:* left SO area	1 mA, 8 cm^2^ (active), 48 cm^2^ (ref), 10 min	0.125	M1-anodal, PMd-anodal, sham (crossover)	M1-tDCS: Enhanced performance and stabilized retention; PMd-tDCS: Attenuated retention
Ehsani et al., 2016	SRTT *right hand*	Mean RT and number of errors of each block	M1 CB	During learning	CL (M1) over CB	M1 *Active:* C3 *Ref:* right SO area CB *Active:* 1 cm below inion *Ref:* over right arm	2 mA, 25 cm^2^, 20 min	0.08	M1-anodal, CB-anodal, sham	Reduced number of errors during learning with CB atDCS, improved RTs and number of errors during retention with both M1 and CB atDCS
Stagg et al., 2011	SRTT, SFTT *right hand*	*SRTT:* ΔRT = meanRT_block_/baselineRT; *SFTT:* ΔRT = meanRT_block_/first sequence RT	M1	During learning (*N* = 7) Before learning (*N* = 8)	CL	*Active:* 5 cm lateral and 2 cm anterior to Cz *Ref:* right SO area	1 mA, 35 cm^2^, 10 min	0.029	Anodal, cathodal, sham (crossover for each Exp.)	*SRTT:* no significant effect on performance induced by tDCS; *SFTT*: tDCS during behavior induced polarity specific modulation of performance, whereas tDCS prior to training led to slower learning with both polarities
Ambrus et al., 2016	SRTT *right hand*	RTs of each block were divided by the RTs of block one	M1	During learning	CL	*Active:* FDI hotspot (TMS) *Ref:* right SO area	1 mA, 35 cm^2^, 12–14 min	0.029	Anodal, cathodal, sham (crossover)	tDCS did not show impact on performance, possibly due to the combination of different interventions (tDCS+TMS)
Wade and Hammond, 2015	SRTT *right hand*	Median RTs of each block were divided by the median RTs of block one, accuracy	PM	During observational learning	CL	*Active:* 2 cm anterior, 2 cm medial from C3 *Ref:* right SO area	1 mA, 24 cm^2^, 14 min	0.042	anodal/sequenced anodal/random sham/sequenced sham/random	atDCS during observational phase improved subsequent performance
Nitsche et al., 2010	SRTT *right hand*	RTs of each block were divided by the RTs of block one	PMd	*Exp. 1:* during REM *Exp. 2:* during learning *Exp. 3:* 4 h after learning, imdtly. before rehearsal	CL	*Active:* 3 cm anterior to C3 *Ref:* above right orbit	1 mA, 35 cm^2^, 15 min	0.029	*Exp. 1*- Group A: anodal, sham or cathodal, sham Group B: anodal, sham *Exp. 2* - anodal, cathodal, sham *Exp. 3* - anodal, sham (crossover)	Improved recall of SRTT if tested immediately after atDCS applied during REM; Evidence for a prominent involvement of PMd in procedural motor memory retention during REM sleep
Saucedo Marquez et al., 2013	SFTT, SVIPT *non-dominant hand*	*SRTT:* Skill index = % correct sequences/mean response time per each 40 s trial *SVIPT:* 1-error rate/error rate(ln(duration)^*b*^)	M1	During all 3 learning sessions	CL	*Active:* FDI hotspot (TMS) *Ref:* IL shoulder	1 mA, 25 cm^2^ (active), 99 cm^2^ (ref), 20 min	0.04	Anodal, sham	Improved SFTT during acquisition and improved SVIPT performance only at retention with atDCS
Saimpont et al., 2016	SFTT *left hand*	Number of correct sequences	M1	During MIm	CL	*Active:* C4 *Ref:* left SO area	2 mA, 35 cm^2^, 13 min	0.057	MIm+anodal, MIm+sham, Read+anodal	Enhanced performance in MIm+atDCS group
Tecchio et al., 2010	SFTT *left hand*	*Performance index* = median execution time of correct series of each block; Accuracy: Number of incorrect sequences per block	M1	Between baseline and re-test	CL	*Active:* C4 *Ref:* IL arm	1 mA, 35 cm^2^, 15 min	0.029	Anodal, sham	Enhanced early retention of the trained sequence by atDCS
Ferrucci et al., 2013	SRTT *bimanually*	Difference in RT between random and sequenced blocks	CB	Between baseline and re-test	Over CB	*Active*: 2 cm below inion *Ref:* right arm	2 mA, 35 cm^2^, 20 min	0.057	Anodal, sham (crossover)	Improved performance after atDCS
Wessel et al., 2016	Sequence learning *right hand*	*Tapping error* (synchronization): absolute time interval where the acoustic cue and the key press did not overlap; *Timing accuracy* (continuation): absolute difference between tapping interval and referring interstimulus interval	CB	During learning	IL	*Active:* 3 cm lateral to the inion *Ref*: right buccinator muscle	2 mA, 25 cm^2^, 20 min	0.08	Anodal, sham (crossover), cathodal (Control group)	Improved performance in the retention-tests of the synchronization part with anodal CB-tDCS
Reis et al., 2009	SVIPT *right hand*	*Skill index* = 1-error rate/error rate(ln(duration)^*b*^)	M1	During all 5 learning sessions	CL	*Active:* APB hotspot (TMS) *Ref:* right SO area	1 mA, 25 cm^2^, 20 min	0.04	Anodal, cathodal, sham	Enhanced total skill acquisition with atDCS compared to sham, effect of atDCS was specific for induction of retention (off-line effects); Improved performance remained at 3 months in the anodal group
Cantarero et al., 2015	SVIPT *right hand*	*Skill index* = 1-error rate/error rate(ln(duration)^*b*^)	CB	During all 3 learning sessions	IL	*Active:* 3 cm lateral to inion *Ref:* right buccinator muscle	2 mA, 25 cm^2^, 20 min	0.08	Anodal, cathodal, sham	On-line learning rather than off-line learning enhanced by CB-atDCS compared to cathodal and sham tDCS
Schambra et al., 2011	SVIPT *bimanually*	*Skill index* = 1-error rate/error rate(ln(duration)^5.424^); baseline skill (mean skill of the 1st 10 trials of block 1) and final skill (mean skill of the last 10 trials of block 6)	M1	During the middle of all 3 sessions	CL	*Active:* either left or right FDI hotspot (TMS) *Ref:* IL deltoid	1 mA, 25 cm^2^, 20 min	0.04	*Right-hand training*: anodal-left M1, anodal-right M1, sham *Left-hand training:* anodal-right M1, anodal-left M1, sham	Left M1-tDCS induced greater skill learning than sham and a trend for greater enhancement than right M1-tDCS
Vollmann et al., 2013	VPFT *right hand*	*Spatial accuracy* (numerical distance between the on-screen force and reference bar, represented as averages of spatial accuracy for 1400 time points of each trial)	SMA preSMA M1	During learning	CL	M1 *Active*: FDI hotspot (TMS) SMA *Active*: indentified with MRI scan pre-SMA *Active*: identified with MRI scan *Ref:* forehead	0.75 mA, 10.7 cm^2^ (active), 100 cm^2^ (ref), 20 min	0.07	Anodal, sham	Improved performance induced by M1 and SMA-tDCS, but not by pre-SMA stimulation
Antal et al., 2004	VM coordination *right hand*	Number of correct tracking movements	V5 M1 V1	During first 2 blocks of learning	CL	V5 *Active:* 4 cm above the mastoid-inion line, 7 cm left of the midline in the sagittal plane *Ref:* Cz V1 *Active:* Oz *Ref:* Cz M1 *Active:* hand area (TMS) *Ref:* right SO area	1 mA, 35 cm^2^, 10 min	0.029	Anodal: V5, M1, V1 Cathodal: V5, M1, V1 No-stim	Improved performance during acquisition induced by M1 and V5 atDCS
Antal et al., 2008	VM coordination *right hand*	Number of correct tracking movements	V5 M1	During first 2 blocks of learning	CL	V5 *Active:* 4 cm above the mastoid-inion line, 7 cm left of the midline in the sagittal plane *Ref:* Cz M1 *Active:* hand area located by TMS *Ref:* right SO area Control (Cz): *Active:* Cz *Ref:* right SO area	1 mA, 35 cm^2^, 10 min	0.029	Anodal: V5, M1, Cz Cathodal: V5, M1, Cz Sham; No-Stim	Performance of movement tracking improved during acquisition after both anodal and cathodal tDCS over both cortical areas
Shah et al., 2013	Ankle VM task *non-dominant leg*	*Accuracy index (AI)* = 100(P–E)/P E = root-mean-square (rms) error between target line and response line; *P* = rms value between sine wave and mid-line separating upper and lower phases; MEP amplitudes	CB M1	During learning	IL (CB) CL (M1)	CB *Active:* 3 cm lateral to the inion *Ref:* IL buccinator muscle M1 *Active:* TA area (TMS) *Ref:* CL forehead	1 mA, 8 cm^2^ (active), 35 cm^2^ (ref), 15 min	0.125	CB-anodal, CB-cathodal, M1-anodal, M1-cathodal, M1-sham (crossover)	Target-tracking accuracy improved by CB-anodal, CB-ctDCS and M1-atDCS, independent from changes in MEP amplitude
Prichard et al., 2014	Continuous word/shape tracing *non-dominant hand*	*Final score* = percentage of correct tracing (perfect match = 100; sum of the difference between trace and template image)	M1	After 1st learning block (for 3 days)	CL	M1-SO *Active:* FDI hotspot (TMS) *Ref:* SO area M1-M1 *Active:* FDI hotspot (right M1) *Ref:* FDI hotspot (left M1)	1 mA, 16 cm^2^, 20 min	0.0625	Anodal, sham	Improved motor skill learning with uni- and bilateral M1-tDCS driven by online learning effects
Naros et al., 2016	Exoskeleton-based tracing *left hand*	Highscore = Σ(i = 2)^*n*^*a*(*n* − 1)−(f1^*^t(n)+f2^*^err(n)) n = N° of reached targets, a(n 1) = score of the last target with a(1) = 1000, t(n) = time to reach the target, err(n) = total deviation from trajectory, f1, f2 weighting factors (f1 = 0.3, f2 = 0.3)	M1	Prior to the learning	CL_anodal_IL_cathodal_	Anodal *Active*: C4 *Ref:* left forehead Cathodal *Active:* C3 *Ref*: right forehead bi-tDCS *Active:* C4 *Ref:* C3 ds-tDCS *Active:* C4 and right forehead *Ref:* C3 and left forehead	1 mA, 16 cm^2^ (active), 35 cm^2^ (ref), 20 min	0.0625	Anodal, cathodal, bi-tDCS, ds-tDCS, sham	Improved final motor performance at the end of training induced only by the two bilateral paradigms
von Rein et al., 2015	Ball rotation *bimanually*	Number of ball rotations/min	M1	During right hand learning with MVF (or watching of stationary left hand)	CL	*Active:* M1—following Montreal Neurological Institute (MNI) coordinates *Ref:* frontal orbit	1 mA, 35 cm^2^ (active), 100 m^2^ (ref), 20 min	0.029	Anodal, sham, Control	Stronger MVF-induced performance with atDCS at Day 1 (online effects) and Day 2 (retention)
Kaminski et al., 2013	Whole-body dynamic balance	*Time in balance* (individual time of each subject to keep the balance platform in a horizontal position as long as possible during the 30 s	SMA PFC	During the first 20 min of learning (Day 1)	SMA over midline, right PFC	Group A *Active(sham):* SMA (MNI-based coordinates) *Ref(sham):* PFC (not specified) Group B *Active:* SMA *Ref*: PFC Group C *Active:* PFC *Ref:* SMA Group D *Active:* SMA *Ref:* PFC (50 cm^2^)	1 mA, 35 cm^2^ (cathode 50 cm^2^ in group D), 20 min	0.029	*Group A* (sham) *Group B* (anodalSMA) *Group C* (cathodalSMA) *Group D* (anodalSMA)	Impaired skill learning on day 1 and 2 with anodal SMA and cathodal PFC; Results possibly due to PFC modulation since control stimulation with larger (more ineffective) on PFC electrode did not affect learning
Zhu et al., 2015	Golf putting task *right arm*	*Number of successful putts* (first and last block of Day 2)	dlPFC	During learning	CL	*Active:* right SO area *Ref:* F3	1.5 mA, 25 cm^2^, 15–20 min	0.06	Cathodal, sham	Enhanced golf putting performance during Training and Test phase with ctDCS
**MOTOR ADAPTATION PARADIGMS**										
Galea et al., 2011	VAT *right arm*	*Angular end point error:* Angle between the line connecting the starting position to the center of the target and the line connecting the starting position to the end point	M1 CB Oz	During 2nd half of pre-adaptation + adaptation	IL (CB) CL (M1) OZ midline	*Exp. 1/2/3—*CB *Active:* 3 cm lateral to the inion *Ref:* right buccinator muscle M1 *Active:* FDI hotspot (TMS) *Ref:* right SO area *Exp. 3—*Oz *Active:* Oz *Ref:* right buccinator muscle	2 mA, 25 cm^2^, 15 min	0.08	*Exp. 1* CB-anodal, M1-anodal, CB/M1-sham *Exp. 2* CB-anodal, M1-anodal, CB/M1-sham *Exp. 3* CB-anodal, OC-anodal	Faster adaptation to visuomotor rotation with CB-tDCS and increased retention with M1-tDCS
Block and Celnik, 2013	VAT *both arms*	*Final angular error:* angular deviation from the target when the cursor was 10 cm from home position	M1 CB	During last baseline block and adaptation	IL/trained CL/untrained	CB *Active:* 3 cm lateral to the inion *Ref:* IL buccinator muscle M1 *Active:* FDI hotspot (TMS) *Ref:* IL SO area	2 mA, 25 cm^2^, 15 min	0.08	*Exp. 1/2* CB-anodal, M1-anodal, CB/M1-sham *Exp. 3* CB-anodal, CB-sham	Faster adaptation with CB-tDCS, but none of the stimulation sites affected intermanual transfer
Herzfeld et al., 2014	Force fields *right arm*	*Hand velocity* perpendicular to the direction of target (cm/s); *Force index:* force produced by subject in an error-clamp trial compared to the ideal force	M1 CB	At onset of 2nd null field + during adaptation	IL (CB) CL (M1)	CB *Active:* 3 cm lateral to the inion *Ref:* Right buccinator muscle M1 *Active:* FDI hotspot (TMS) *Ref:* right SO area	2 mA, 25 cm^2^, 25 min	0.08	*CB:* anodal, cathodal, sham *M1:* anodal	Increased rate of learning with CB-atDCS; Impaired ability to respond to sensory feedback and decreased rate of learning with CB-ctDCS; M1-atDCS had no effect on these variables; Neither CB nor M1-tDCS altered stabilization processes of motor memory; Retention impaired by CB-ctDCS and unaffected by M1-tDCS
Taubert et al., 2016	Force fields *right arm*	*Reaching error:* perpendicular displacement of the hand trayectory in cm from a straight line joining start and target point (300 ms) after movement start	CB	During learning of 1st force field	IL	*Active:* 2 cm below inion *Ref:* right buccinator muscle	2 mA, 25 cm^2^, 20 min	0.08	Anodal, cathodal, sham	CB-tDCS induced impairments in short-term retention during initial acquisition of a task A and performance deficits in the re-acquisition session (24 h later); Interference task B unaffected
Orban de Xivry et al., 2011	Force fields *right arm*	*Adaptation index (AI)*: Ratio between measured and ideal force taken at the time of peak velocity ^*^ 100; *Generalization index*: AI (T2 or T3)/ AI (T1) ^*^100 (at the end of learning); *T* = target	M1 PPC	During adaptation	CL	M1 *Active:* FDI hotspot (TMS) PPC *Active:* P3 *Ref:* right SO area	1 mA, 25 cm^2^, 20 min	0.04	M1-anodal M1-cathodal M1-sham PPC-anodal PPC-cathodal	M1-tDCS had no effect on adaptation patterns during learning, but increased generalization in intrinsic coordinates but not extrinsic coordinates; tDCS over PPC had no effect on learning or generalization
Hunter et al., 2009	Force fields *right arm*	*Summed error:* cumulative perpendicular distance between the hand position and the ideal trajectory for the duration of reaching	M1	During adaptation	CL	*Active:* biceps hotspot (TMS) *Ref:* right SO area	1 mA, 35 cm^2^, 17 min	0.029	Anodal, sham (crossover)	Greater global reaching (overshoot) error during early stage of de-adaptation with atDCS
Panouillères et al., 2015	Saccadic adaptation (backward and forward)	*Saccadic gain change* = (Gain saccade n—mean gain Pre10 min)/mean gain Pre10 min; same for changes in duration and peak velocity	CB	After 1st pre-adaptation until end of post-adaptation	Midline	*Active:* centered over the inion *Ref:* over superior aspect of the right trapezius muscle	2 mA, 35 cm^2^, 25 min	0.057	Anodal, cathodal, sham	Faster forward and backward adaptation with ctDCS, as well as increased velocity in forward adaptation; Strongly impaired forward adaptation with atDCS, and reduced velocity in backward adaptation
Panico et al., 2016	PAP *right arm*	*Deviation:* Distance between the point touched by the subject and the actual position of the target on the horizontal and vertical axes (index of accuracy)	CB	During adaptation	IL	*Active:* Right deltoid muscle *Ref*: 1 cm below and 3 cm right to the inion	2 mA, 25 cm^2^, 16 min	0.08	Cathodal, sham	Larger rightward deviation during exposure to prisms and a larger leftward deviation after removal on the horizontal axis with ctDCS
Jayaram et al., 2012	Split-belt walking	*Step symmetry* = (step length[fast]—step length[slow]/step length[fast] + step length[slow])	CB	During adaptation	IL to fast leg IL to slow leg	*Active:* 3 cm lateral to the inion *Ref:* IL buccinator muscle	2 mA, 25 cm^2^, 15 min	0.08	Anodal(fast) cathodal(fast) anodal(slow) cathodal(slow) sham	Locomotor adaptation improved with atDCS, and slowed down with cerebellar ctDCS IL to the fast leg
**USE-DEPENDENT LEARNING (UDL) PARADIGMS**										
Rosenkranz et al., 2000	RTM *right thumb*	*Angular deviation* of training and post-training movements from pre-training movements	M1	During last 5 min of training	CL	*Active:* APB hotspot (TMS) *Ref:* right SO area	1 mA, 35 cm^2^, 5 min	0.029	Anodal, cathodal, no-tDCS (crossover)	Reduced angular deviation with anodal and ctDCS during 10 min post-training, indicating an interference of tDCS with repetitive-based plasticity processes
Galea and Celnik, 2009	RTM *right thumb*	Percentage of TMS-evoked thumb movements falling within the training target zone; TMS-evoked movement direction distance relative to training direction (degrees); mean magnitude of first-peak acceleration in the extension/flexion direction; MEP peak-to-peak amplitudes	M1	During training	CL	*Active:* APB hotspot (TMS) *Ref:* right SO area	1 mA, 25 cm^2^, 30 min	0.04	Anodal, sham, cathodal (crossover)	Enhanced retention of motor memories with atDCS reflected by: changes in all kinematic measures, longer-lasting effects relative to training alone, required association of training and stimulation, and polarity specificity
Cabral et al., 2015	RTM *right thumb*	*MEP* peak-to-peak amplitude (baseline and postsession)	M1	Before, during, or after training (counterbalanced)	CL	*Active:* FDI hotspot (TMS) *Ref:* right SO area	1 mA, 35 cm^2^, 13 min	0.057	Anodal, sham (crossover)	Increased corticospinal excitability when atDCS was applied before the motor task
Koyama et al., 2015	RTM *left thumb*	Peak *acceleration* of movement	M1	During training	CL	*Active:* right M1 *Ref:* left M1 (based on T1 anatomical image)	1 mA, 25 cm^2^, 25 min	0.04	Anodal, sham	Improvement of peak acceleration at 24 h (retention) after atDCS compared to sham
Rroji et al., 2015	RTM *non-dominant thumb*	*Performance improvemen*t (%) = (peak velocity _1…10block_/ block1) ^*^ 100	M1	During training	CL	*Active:* ABP hotspot (TMS) *Ref:* IL shoulder	1 mA, 25 cm^2^ (anode), 99 cm^2^ (cathode), 20 min	0.04	Anodal, sham (crossover)	Retention performance (1 week after training) was improved with atDCS

*The table describes the main outcome measure, stimulation parameters and most important key findings from each study. The studies are ordered as they appear in the in-text references. APB, abductor pollicis brevis muscle; atDCS, anodal transcranial direct current stimulation; CB, cerebellum; CL, contralateral; ctDCS, cathodal transcranial direct current stimulation; dlPFC, dorsolateral prefrontal cortex; FDI, first dorsal interosseus muscle; IL, ipsilateral; J, current density; Lpfc, lateral prefrontal cortex; M1, primary motor cortex; MEP, motor evoked potential; MIm, motor imagery; mPFC, medial prefrontal cortex; MRI, magnetic resonance imaging; MVF, mirror visual feedback; PAP, prism adaptation procedure; PFC, prefrontal cortex; PM, premotor cortex; PMd, dorsal premotor cortex; PPC, posterior parietal cortex; REM, rapid eye movement sleep; ROI, region of interest; RT, reaction time; RTM, repetitive thumb movement; SFTT, serial finger tapping task; SMA, supplementary motor area; SO, supraorbital area; SRTT, serial reaction time task; SVIPT, sequential visual isometric pinch task; TA, tibialis anterior muscle; TMS, transcranial magnetic stimulation; V1, primary visual cortex; V5, extrastriate visual area; VAT, visuomotor adaptation task; VM, visuomotor; VPFT, visuomotor pinch force task*.

### Modulating skill learning

Skill learning refers to a process that results in improving the trade-off between speed and accuracy (Reis et al., 2009), typically achieved by reducing movement variability (Smuelof et al., 2012). Investigations have used tDCS to either modulate learning or to better understand the underlying learning processes (Orban de Xivry and Shadmehr, 2014; Savic and Meier, 2016). However, the number of brain regions involved in skill learning is vast (Ungerleider et al., 2002) which has led to various targeted brain regions for tDCS application, electrode montages, and types of motor tasks. The leading paradigms combined with tDCS are motor sequence tasks, including serial reaction time task (SRTT), sequential finger tapping tasks (SFTT), and sequential visual isometric pinch task (SVIPT) (see Table 2 for details).

**Table 2:** **Characterization of the main motor paradigms described in this mini-review**.

**Motor task**	**Description**
SRTT	Participants respond to visual cues presented on a screen by pressing an associated keyboard response. The position of the visual cue is either presented in a repeating sequence or random.
SFTT	A specific order of sequence elements is presented on a screen that present specific finger movements. Participants are instructed to make the representative key-presses as fast and accurate as possible.
SVIPT	Participants control the movement of a cursor displayed on a computer screen by squeezing an isometric force transducer using the thumb and index finger. The aim is to move the cursor as quickly and accurately as possible between the start position and a numbered order of target zones. The magnitude of pinch force applied to the sensor is non-linearly (usually a logarithmic transduction is applied) related to the displacement of the cursor.
VPFT	Similar to the SVIPT, participants match their own pinch force visually displayed by a force bar on a computer screen with the height of a moving reference bar by squeezing a force transducer.
VAT	Participants make hand-reaching movements with a pen over a horizontal digitizing tablet to respond to a target displayed on a vertical screen. Vision of the hand was not visible to participants, but a cursor on the screen was given to participants to represent the position of their hand. Participants are instructed to make rapid and straight uncorrected movements throughout training. After some practice, a perturbation is introduced by applying a visual rotation (e.g., by 30° counterclockwise) of the cursor. Participants adapt incrementally their movements to the new position and show large and prolonged after-effects once the perturbation is removed.
Force fields	Participants hold a robotic arm handle in order to make reaching movements to a specific target displayed on a screen. Vision of the hand was obstructed, however, visual feedback of hand position is provided on the screen. After baseline performance, reaching is perturbed by a force field that pushes the hand perpendicular to the direction of movement. After participants adapt to the force field perturbation, participants show large after-effects when the perturbation is removed.

*SFTT, sequential finger tapping task; SRTT, serial reaction time task; SVIPT, sequential visual isometric pinch task; VAT, visuomotor adaptation task; VPFT, visual pinch force task*.

Several studies have reported enhanced SRTT performance and retention with simultaneously applying atDCS over M1. This is shown by reduced reaction times (RTs), a common way to quantify sequence acquisition (Nitsche et al., 2003; Kang and Paik, 2011; Kantak et al., 2012; Ehsani et al., 2016). Comparably reduced RTs were found during the recall of a sequence task when tDCS was applied over premotor (PM) cortex throughout REM sleep (Nitsche et al., 2010). A few studies, however, have presented null effects of tDCS on RTs, specifically when stimulation was not applied during training (Stagg et al., 2011), or when tDCS was combined with single-pulse TMS, causing a potential reduction of tDCS' efficacy (Ambrus et al., 2016). Moreover, when tDCS was applied over PM during SRTT, neither acquisition nor consolidation was modulated (Nitsche et al., 2003), but instead interfered with the retention of learned sequences (Kantak et al., 2012). In contrast, when PM-tDCS was applied while participants watched a video of a hand performing key-press sequences prior to training, RTs were reduced in comparison to sham stimulation. This suggests that increasing excitability of a region involved in action observation promotes skill acquisition (Wade and Hammond, 2015). Additional studies have revealed significant benefits of tDCS on SFTT learning. Interestingly, the number of correctly executed sequences increased both when M1-tDCS was applied concurrently with performance (Saucedo Marquez et al., 2013), and when tDCS was applied during motor imagery of sequences (Saimpont et al., 2016). When individuals received M1-atDCS during performance, RTs decreased during training (Stagg et al., 2011), whereas when M1-atDCS was applied between two training sessions, reduced execution time of correct sequences was found during early consolidation (Tecchio et al., 2010), i.e., stabilization of the motor memory rapidly after its initial acquisition (Brashers-Krug et al., 1996). Together, this suggests M1 as an important site for storage of motor sequences. On the other hand, the role of the cerebellum, a structure critical for motor adaptation (Tseng et al., 2007; Donchin et al., 2012; Izawa et al., 2012), is not well understood for procedural sequence learning (Jenkins et al., 1994; Doyon et al., 2002; Shimizu et al., 2016). Only a few studies have addressed the effects of cerebellar atDCS on sequence learning. For example, cerebellar stimulation applied during SRTT performance reduced the error rate (Ehsani et al., 2016), whereas it reduced RTs when applied prior to a follow-up session (Ferrucci et al., 2013). Interestingly, both M1 and cerebellar atDCS showed enhanced retention of SRTT performance (Ehsani et al., 2016). In a different type of sequence learning which relies on lateral cerebellar function, atDCS over cerebellum reduced tapping movement errors in follow-up sessions. Thus, it appears cerebellar tDCS may facilitate retention of complex motor skills (Wessel et al., 2016).

Simultaneously applying M1-atDCS during SVIPT learning facilitated skill acquisition over several consecutive days of training (Reis et al., 2009; Schambra et al., 2011; Saucedo Marquez et al., 2013). Specifically, stimulation promoted between-session (Reis et al., 2009) or long-term retention processes (Saucedo Marquez et al., 2013). Interestingly, when atDCS was applied over the cerebellum, skill acquisition was enhanced within-session (online) rather than between-session gains. Here, skill improvement was marked by lower error-rates rather than movement time (Cantarero et al., 2015). In a slightly different task (visuo-motor pinch force task, see Table 2 for details), tDCS over secondary motor areas such as the supplementary motor area (SMA) showed to increase participants' spatial accuracy, providing new insights about the role of SMA during skill performance (Vollmann et al., 2013).

Beyond the SRTT, SFTT, and SVIPT tasks, there are additional investigations with varying tasks that have explored tDCS effects during skill learning. For instance, atDCS applied either over M1 or an extrastriate visual area *during* a visuo-motor coordination task improved early performance of correctly tracked movements (Antal et al., 2004), whereas performance was enhanced for both tDCS polarities when stimulation was applied *prior* to training (Antal et al., 2008). Moreover, both uni-lateral and bi-lateral M1-tDCS applied concurrently with skill tracing tasks showed enhanced target-tracking accuracy (Shah et al., 2013; Prichard et al., 2014; Naros et al., 2016), an effect similarly found when pairing training with anodal and cathodal cerebellar tDCS (Shah et al., 2013). Furthermore, combining mirror visual feedback with M1-atDCS improved performance of a manual ball-rotation task with the untrained hand, likely due to additive effects on motor performance (von Rein et al., 2015). Accordingly, when the anode electrode was placed over SMA and cathode over right prefrontal cortex (PFC) performance of a dynamic whole body task was impaired (Kaminski et al., 2013). On the other side, PFC-ctDCS improved performance of a golf-putting task during acquisition and retention, highlighting a promising application of tDCS toward everyday motor activities (Zhu et al., 2015).

### Modulating motor adaptation

Another type of learning studied in laboratory settings is motor adaptation, or a reduction of errors in response to environmental changes via generating an internal model to predict the consequences of actions. Adaptation is generally tested in a variety of error-based tasks (prisms, rotations, force fields), where quickly accounting for perturbations leads to large behavioral changes (Krakauer and Mazzoni, 2011). In relation to brain stimulation, a recent study applied tDCS to distinct brain regions while participants learned a visuomotor rotation (see Table 2 for details). Specifically, they found cerebellar atDCS resulted in faster reduction of errors caused by a consistent visuomotor-rotation (Galea et al., 2011; Block and Celnik, 2013), whereas atDCS over M1 showed a marked increase in retention of the newly learned rotation (Galea et al., 2011). By using tDCS, this study was able to show an important dissociation in acquisition and retention processes related to motor adaptation and further highlighted the distinct roles of the cerebellum and motor cortex. Furthermore, tDCS over these regions did not enhance intermanual transfer of visuomotor rotation learning (Block and Celnik, 2013) suggesting that these structures do not play as critical of a role for this process.

Another study tested tDCS over cerebellum and M1 during force-field adaptation (see Table 2 for details) and consistent with the results reported by Galea et al. (2011), the authors found that cerebellar atDCS enhanced the rate of acquisition (Herzfeld et al., 2014). This study also showed that cerebellar ctDCS delayed the feedback response to the introduced perturbation and decreased the learning rate. Taubert et al. (2016) observed impaired adaptation and re-acquisition of a force-field perturbation with cerebellar atDCS, while no effect was found for ctDCS. It is possible that the experimental design differences of these studies may explain the inconsistent findings.

Regarding the role of M1 in force-field adaptation, M1-tDCS did not alter the rate of adaptation learning during reaching movements (Orban de Xivry et al., 2011; Herzfeld et al., 2014) similar to visuomotor adaptation. While most studies have reported that motor adaptation is not affected by M1-tDCS, one study showed atDCS over M1 biceps brachii representation led to greater overshooting errors in force-field learning once the field was removed, suggesting a possible role of M1 in the adaptation process of reaching movements (Hunter et al., 2009). While these results remain inconclusive, M1-tDCS showed a clear increase of generalization in intrinsic coordinates for joints and muscles during force-field adaptation, without changing extrinsic generalization patterns. In contrast, tDCS tested over posterior parietal cortex had no effects on learning or generalization (Orban de Xivry et al., 2011).

A few studies have also used tDCS to examine functions of the cerebellum outside of visuomotor and force-field adaptation. One study showed that cerebellar excitability plays a crucial role in saccadic adaptation (Panouillères et al., 2015), as well as in all stages of prism adaptation, i.e., in flexible motor adjustments in response to changes of the visual field (Panico et al., 2016). Moreover, Jayaram et al. (2012) were able to modulate locomotor adaptation by applying tDCS over the cerebellum while participants walked on a split-belt treadmill at two different speeds. They found atDCS ipsilateral to the fast leg accelerated adaptation (i.e., promoted faster gait step-symmetry), whereas ctDCS slowed adaptation. Interestingly, atDCS effects primarily affected spatial, rather than temporal components of walking (Jayaram et al., 2012).

### Modulating use-dependent learning

Use-dependent learning (UDL) describes a phenomenon where short-term motor memories are formed and retained due to repeatedly trained motor actions, thus inducing representational changes in the motor cortex (Classen et al., 1998). Rosenkranz et al. (2000) first addressed the effects of tDCS over M1 on UDL by comparing the directional variation of TMS-induced thumb movements (opposite to the trained direction) before and after tDCS application. They found that applying tDCS during the last 5 min of 30-min thumb-movement training resulted in smaller TMS-induced angular deviation compared to controls. In other words, anodal or cathodal tDCS with training produced a movement direction similar to the pre-training direction, whereas movements of the control group were biased to the trained direction. The authors concluded that tDCS preserves pre-training cortical movements by interfering with the mechanisms of UDL and the formation of motor memories (Rosenkranz et al., 2000). In contrast, Galea and Celnik (2009) demonstrated enhanced retention effects of repetitive thumb training when atDCS over M1 was applied throughout the 30 min training period. Importantly, cathodal and sham group responses did not show significant changes. The inconsistencies between these two studies could potentially be explained by the different stimulation periods of tDCS (5 vs. 30 min). On the other hand, the prior state of the system (i.e., 25 min of training vs. no training) may not be the same when tDCS is applied at training onset vs. at the end of training (Galea and Celnik, 2009). A recent study aimed to determine whether M1-tDCS applied before, during, or after motor training enhances UDL. The authors found larger MEP amplitudes (first dorsal interosseous muscle) only when atDCS was applied *before* motor training. This suggests tDCS prior to training benefits optimization of UDL (Cabral et al., 2015). However, these results are inconsistent with other studies. Galea and Celnik (2009) showed a significant effect on training by applying tDCS *during* the training, an effect that is similarly found with sequence-learning (Stagg et al., 2011). Furthermore, recent results showed enhanced retention of ballistic thumb movements when M1-atDCS was applied *during* training when evaluating both peak velocities and accelerations of thumb movements (Koyama et al., 2015; Rroji et al., 2015).

## Considerations on motor learning modulation and new perspectives

Overall, the results summarized in this review highlight the need for new stimulation paradigms based on more natural and individualized stimulation protocols, aiming to optimize the desired stimulation effects. Variability and contradictions between studies need to be considered, however, this is frequently caused by methodological differences (Paulus, 2011; Horvath et al., 2014, 2015). When considering that different brain regions are likely involved in distinct motor learning processes (Shmuelof and Krakauer, 2011; Penhune and Steele, 2012), the simultaneous (or sequential) electrical stimulation of these areas on the proper polarity and intensity could potentially optimize tCS effects. In this regard, bilateral M1 combined with PFC stimulation has been successfully applied (Vines et al., 2008; Mordillo-Mateos et al., 2012; Leite et al., 2013; Naros et al., 2016). However, the characterization of the effects associated to concomitant stimulation of different brain regions is nearly absent in the literature (Kaminski et al., 2013; Minichino et al., 2015) due to the low focality inherent to the technique and the inability from traditional tDCS devices to simultaneously control multiple stimulation electrodes. Indeed, there has been some progression in recent years. Thus, multifocal tDCS devices using several small-size electrodes (Ruffini et al., 2014), High-Definition tDCS (HD-tDCS) scalp montage (4 × cathode, surrounding a single central anode, Edwards et al., 2013), or concentric electrodes (Bortoletto et al., 2016) provide evidence for more focal tDCS. On the other hand, new devices allowing for EEG recording during simultaneous tDCS also present an excellent tool for the development of individualized stimulation protocols based on the observed individual brain activity (Schestatsky et al., 2013).

Although more investigations are needed to provide a better understanding of the effects induced by tDCS, its impact on motor learning and use for exploring neural substrates underlying motor learning have been successfully demonstrated. In other words, the potential of this technique for basic studies and future clinical treatments seems promising. However, ethical considerations using tDCS for high-performance sports are still a matter of discussion (Reardon, 2016).

## Author contributions

CA and JM contributed to the initial draft, CA, DS, and JM edited the text and wrote the final version of the mini-review.

## Funding

This work was funded by the Spanish MINECO-FEDER (BFU2014-53820-P).

### Conflict of interest statement

The authors declare that the research was conducted in the absence of any commercial or financial relationships that could be construed as a potential conflict of interest.

## Abbreviations

atDCS: anodal transcranial direct-current stimulation
BDNF: brain-derived neurotrophic factor
ctDCS: cathodal transcranial direct-current stimulation
GABA_A_: γ-aminobutyric acid type A
HD-tDCS: high-definition transcranial direct-current stimulation
M1: primary motor cortex
MEP: motor evoked potential
NMDA: N-methyl-D-aspartate
PFC: prefrontal cortex
PM: premotor area
RTs: reaction times
SFTT: serial finger tapping tasks
SMA: supplementary motor area
SRTT: serial reaction time task
SVIPT: sequential visual isometric pinch task
tDCS: transcranial direct-current stimulation
TMS: transcranial magnetic stimulation
UDL: use-dependent learning.
